# Projected incremental changes to extreme wind-driven wave heights for the twenty-first century

**DOI:** 10.1038/s41598-021-87358-w

**Published:** 2021-04-23

**Authors:** J. G. O’Grady, M. A. Hemer, K. L. McInnes, C. E. Trenham, A. G. Stephenson

**Affiliations:** 1 CSIRO Oceans and Atmosphere, Melbourne, Victoria Australia; 2 CSIRO Oceans and Atmosphere, Hobart, Tasmania Australia; 3 CSIRO Oceans and Atmosphere, Canberra, Australian Capital Territory Australia; 4 DATA61 CSIRO, Melbourne, Australia

**Keywords:** Projection and prediction, Physical oceanography

## Abstract

Global climate change will alter wind sea and swell waves, modifying the severity, frequency and impact of episodic coastal flooding and morphological change. Global-scale estimates of increases to coastal impacts have been typically attributed to sea level rise and not specifically to changes to waves on their own. This study provides a reduced complexity method for applying projected extreme wave changes to local scale impact studies. We use non-stationary extreme value analysis to distil an incremental change signal in extreme wave heights and associate this with a change in the frequency of events globally. Extreme wave heights are not projected to increase everywhere. We find that the largest increases will typically be experienced at higher latitudes, and that there is high ensemble model agreement on an increase (doubling of events) for the waters south of Australia, the Arabian Sea and the Gulf of Guinea by the end of the twenty-first century.

## Introduction

Episodic extreme wind-wave events are relevant for probabilistic storm event design studies, to investigate the capacity of engineered infrastructure or natural environments to withstand the harsh action of the ocean, now and into the future. Increasing evidence suggests global climate change will alter wind sea and swell waves, modifying the severity and frequency of episodic coastal flooding and morphological change, exacerbating (or ameliorating) the impacts of sea level rise (SLR)^1^.

Coastal flooding studies including the effect of wind waves have attributed global sea level rise with increases in the frequency of episodic coastal flooding globally using stationary baseline wave climates^2–4^ and non-stationary (baseline and future) wave climates^5,6^. Vousdoukas et al.^5^, investigate the influence of SLR combined with storm surge and wave contribution, but do not specifically detail the contribution of a changing wave climate on future extreme sea level. Melet et al.^7^, consider the low-frequency (mean) changes in sea level from the projected changes in waves. Extreme wave heights on their own are not projected to increase everywhere^8^ and the relative effect of changes to extreme waves rather than SLR on the frequency of coastal impacts has not been shown. The reduced intensity of episodic extreme wave heights could, for example ameliorate the coastal flooding effect of SLR, reduce the flushing of stagnate coastal waters^9^ and/or reduce the depth of closure that relates to the transport of offshore sediments to the surf zone during episodic events, and therefore sediment availability to replenish and protect coastlines between storms^10^.

There is great uncertainty in extreme wave projections due to which future Representative Concentration Pathway (RCP) will play out as a result of future greenhouse gas emissions, government policies, and technological advances^11^. There is also significant uncertainty in global climate model (GCM) internal accuracy (due to model resolution and parametrisation) and the significance/robustness of climate statistics, particularly for rare events (due to limited data in a changing climate). To address the RCP uncertainty, scientific institutions run GCMs for multiple RCPs to span possible futures, and to address climate sensitivity and model uncertainties multiple scientific institutions contribute simulations from their own GCMs to projects such as the Coupled Model Intercomparison project (e.g., CMIP5; CMIP6). The CMIP experiments have enabled the Coordinated Ocean Wave Climate Project^12^ to explore robustness and source of uncertainties associated with projected twenty-first century changes in wind-wave climate, amongst an ensemble of Global Wave Climate Models (GWCM)^13,14^ .

**Figure 1:**
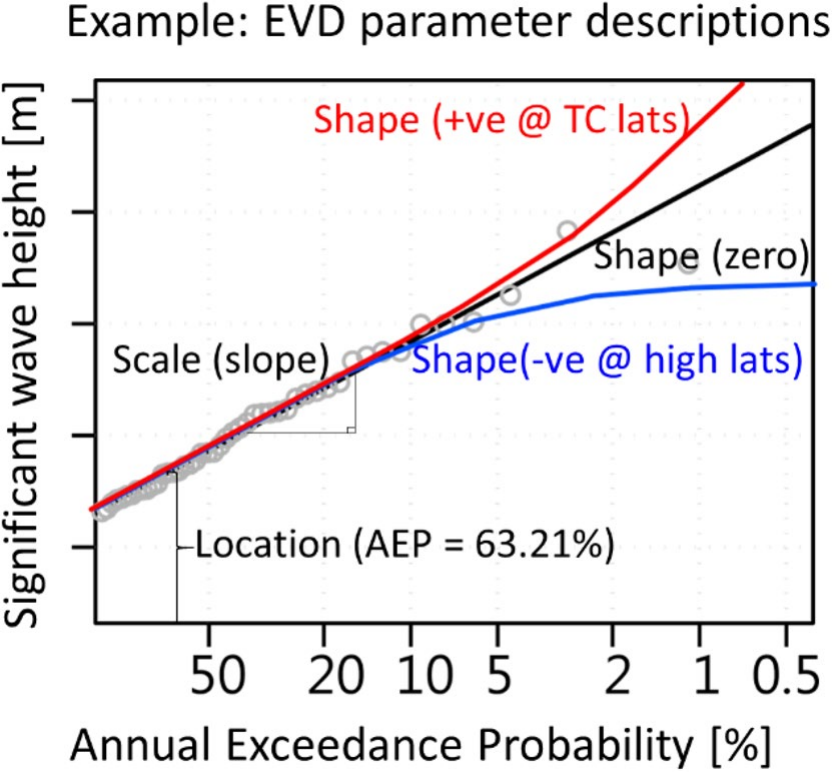
Idealised return level diagram describing the EVD location, scale and shape parameters. Red curve is for a GEV distribution with a positive (+ve) shape parameter typically found at TC latitudes, the black curve is for a Gumbel distribution (zero shape parameter) and the blue curve is for a GEV distribution with a negative (−ve) shape parameter typically found at high latitudes. Grey circles represent pseudo empirically-ranked annual maximum Annual Exceedance Probability (AEP). Created using the R statistical software version 4.0.2^22^.

Extreme value analysis (EVA) provides an avenue to develop extreme value distributions (EVDs) to investigate the probability of rare and extreme episodic events and how they may change^15–17^. A three parameter EVD fit is described by the location, shape and scale parameters, each of which influence the representation of the EVD and consequently the return level plot (Fig. 1). On the return level curve, with exceedance probabilities plotted on a logarithmic x-axis, the location parameter describes the vertical offset, the scale parameter is the log-linear gradient (slope), and the shape parameter describes the curvature of the return level curve. It has been shown that at TC locations the shape parameter is positive, and at high latitudes the shape parameter is negative^18^.The shape parameter describes the behaviour of the highest recorded return levels, with larger shape parameters giving heavier tailed distributions. For a fixed sample size the scale and shape of a distribution is more difficult to estimate than the location, and therefore robust estimates are more attainable for the location parameter of the GEV distribution than the scale or shape parameters. Characteristics of projected twenty-first century change in frequency or intensity of wind-wave events will be represented by changes in these parameters. The shape parameter is most sensitive to the highest recorded return levels. Thus, robust estimates of the shape parameter requires many decades of data, and detecting any changes, from a baseline to future climate, requires notionally twice as much data.

To address statistical significance of rare wave events in a changing climate, different techniques have been used to gain robustness of extreme estimates. These include non-stationary EVD parameter fitting^18–20^, methods for optimal EVD fitting^6^ and the pooling of multi-model data^8^. Wind-wave climate EVA could also benefit from comparing the fit of different EVDs, as has been carried out in studies for extreme sea levels (e.g. Kirezci et al.^4^; Wahl et al.^21^). By their very nature of being rare, a goodness of fit test of empirical data to EVD will always struggle to definitively confirm one modelling technique over another, and therefore model suitability relies on user and community acceptance of model techniques and assumptions.

Downscaling is a process that brings global scale projections of the future climate to the local scale^23,24^. The application, or downscaling, of extreme wave conditions to coastal impact modelling can be resource intensive^5,25^. It has been suggested that reduced complexity methods are required to take the globally-resolved simulation of future wind sea and swell waves to investigate the probabilistic impacts at the local nearshore scale^25^.

This paper will investigate non-stationary EVD fitting to a global wave hindcast and an ensemble of eight GWCMs. Using all available GWCM model years, and assuming a non-stationary location parameter (representative of climate driven change), we establish single-optimal (stationary) estimates of the scale and shape parameters (valid over the long-term—towards centennial time-scale) for each GWCM. After demonstrating that GWCMs are able to replicate the behaviour of the hindcast simulated extreme wave events through EVD parameters, we introduce a simple reduced complexity change factor (CF) downscaling method. This non-stationary EVD method presents, for any location, low, mid and high end-ensemble estimates of future wave heights, as an incremental change relative to baseline extreme wave heights ($\delta {H}_{m0}$).

## Results

### Extreme value distribution behaviour

We compare the global pattern of both the generalised extreme value (GEV) EVD, which has a non-zero shape parameter, with the Gumbel EVD which has a shape parameter of zero, with both fitted to annual maximum values^15^. A hindcast is used as a validation benchmark for an ensemble of eight global wave models forced with independent GCM surface winds^26^. In all EVD modelling a non-stationary location parameter is used. The EVD modelling makes use of all available years of the 37 year hindcast and the 66 year GWCMs datasets (i.e. the combination of the 26 year baseline and two 20-year future time slices for the mid-century and end of century periods) for each of the two different RCPs simulations (RCP 4.5 and RCP 8.5), unless otherwise stated. Results are presented for the low, mid and high-end range of estimates, represented by the 5th, 50th, and 95th quantiles respectively, to demonstrate the uncertainty range of the benchmark hindcast and GWCM ensemble (see “Methods” section).

**Figure 2:**
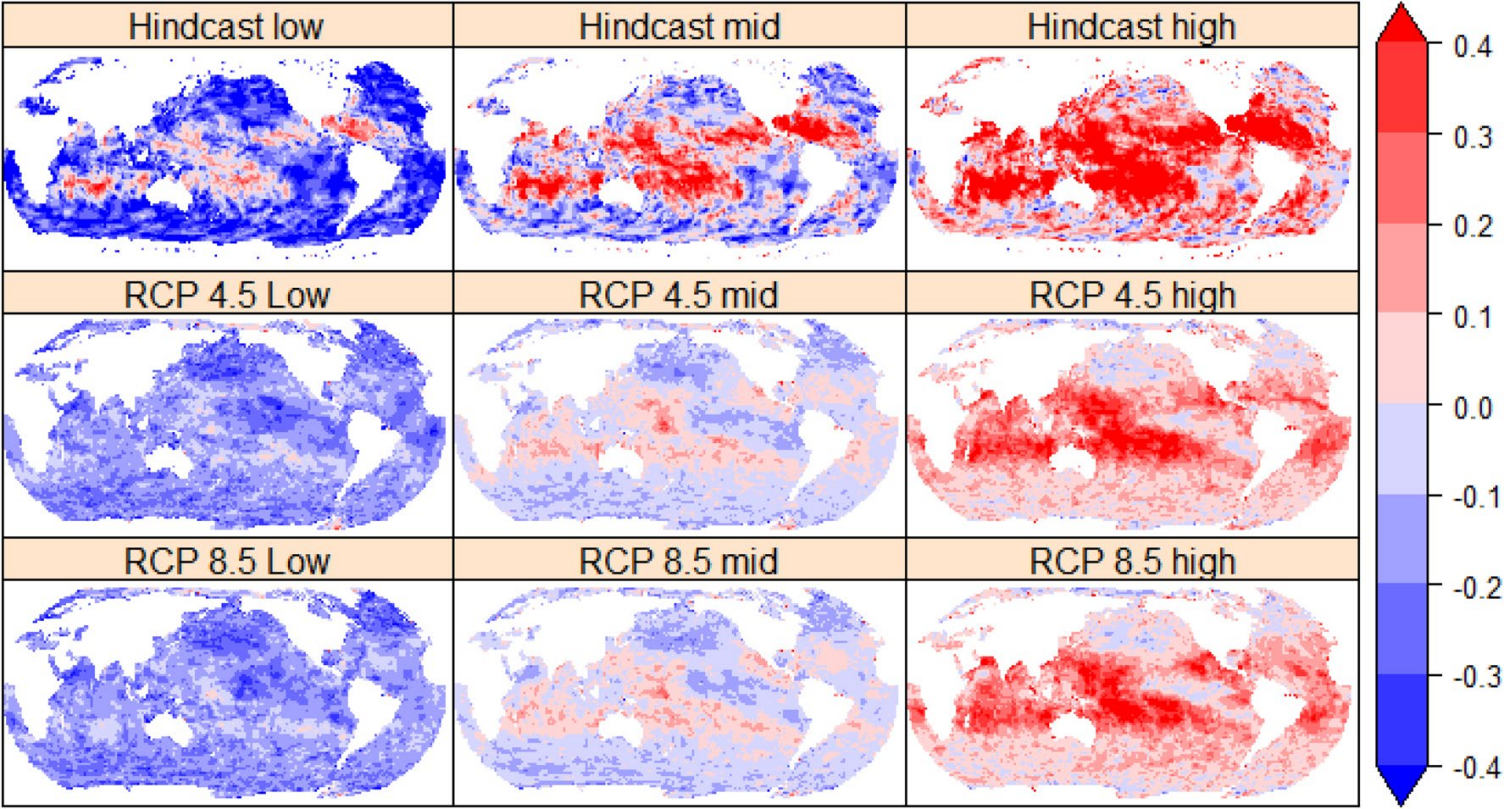
Estimated range of the GEV shape parameter for the full periods of the hindcast and GWCM period (combined baseline, mid and end of twenty-first Century periods) of the two RCP scenarios. Top row: hindcast. Middle: the entire GWCM RCP 4.5 period. Bottom: same as middle but for RCP 8.5. See "Methods" section for definition of estimated ranges: low, mid and high represented by the 5th, 50th, and 95th quantiles respectively. Created using the R statistical software version 4.0.2^22^.

We find that the ensemble range of the EVD parameters fitted to the GWCMs have noticeably similar global patterns to the span of the benchmark hindcast estimates (Figure 2). This indicates some support for the GWCMs ability to represent the general nature of extreme wave events given the limitations on GWCM and forcing resolution and physics. Following global satellite analysis^18^, the hindcast and GWCM mid-range estimates indicate: (1) positive shape parameters at most tropical cyclone (TC) sites, resulting in unbounded annual exceedance probability (AEP) estimates, and (2) typically negative shape parameters at high latitudes, resulting in bounded AEP estimates (Figure 2). Overall, the estimates of the shape parameter in the GWCM are lower in magnitude compared to the hindcast, likely due to the limitations of GWCMs (e.g. grid resolution and parameterised forcing physics) to fully represent the severity, intensity or occurrence of extremes^27,28^. In Figure 2 we start by focusing on the GEV shape parameter as it can be considered the most sensitive parameter to the amount of data available, particularly in a nonstationary climate^19^. Figure 2 shows that the two divergent future RCP climate simulations (RCP4.5 and RCP8.5) have indistinguishable similarity in the shape parameter when considering the unavoidable spatial variability (noise) due to the sampling of passing storms for the two different simulations. The maximum likelihood estimate (MLE) GEV shape parameter for each individual GWCM for the RCP 8.5 simulation is shown in the supplementary report (Figure S1).

Unlike the hindcast, the low end GWCM ensemble estimates indicate that not all models predict a positive shape parameter at latitudes where TCs occur (Fig. 2). This is consistent with the work of Shimura et al.^28^, who found that across this same ensemble of GWCMs, representation of TC driven wave extremes was variable by model in the Western North Pacific Ocean. Due to the low probability of TC occurrence and underestimated intensity, future work is required to better represent the intensities of a larger sample of TC extremes, e.g. via centennial high resolution GWCM or synthetic simulations, to get a better understanding of shape parameter behaviour^28–30^.

The high-end GWCM ensemble estimates indicate that not all models, nor all hindcast estimates, indicate a negative shape parameter at high latitudes (Fig. 2). The hindcast shape parameter in the Southern Ocean alternates in stripes of positive to negative values across the Southern Ocean. Individual GWCM also show this striping (Figure S1). We believe this striping is evidence of limited sample of storm tracks in the dataset.

The fitting to the Gumbel EVD shows large scale parameters in the East and South China seas in the hindcast, which is represented in only a few GWCMs (Figure S2). As with the GEV shape parameter described above, the two divergent future RCP climate simulations (4.5 and 8.5) have indistinguishable similarity in the Gumbel scale parameter, suggesting our assumption of a stationary fitting of the scale parameter for a changing climate is also sensible (Figure S3). It is worth noting here that when the return levels (RL) derived with the Gumbel distribution are compared to those derived with a strong positive GEV shape parameter (typical of TC locations), the resulting difference for the 10% AEP RL is small, however for the 1% AEP event the Gumbel EVD has a 40% lower estimate (Figure S4).

### Climate change-driven alteration of extreme wave heights

**Figure 3:**
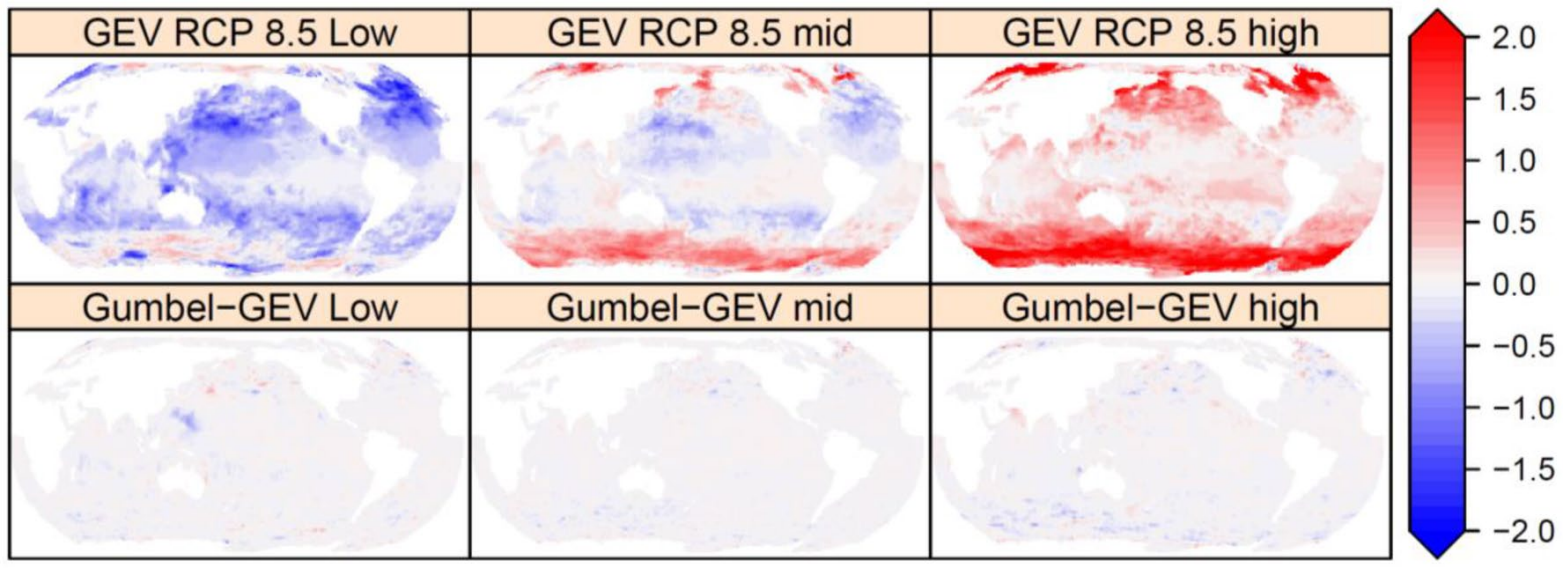
Estimated range of the incremental change in extreme wave height ($\updelta {\mathrm{H}}_{\mathrm{m0,100}}$) [m] for the RCP 8.5 simulations over a period of 100 years. Top row GEV RCP 8.5 and bottom row the difference between Gumbel and GEV RCP 8.5. See "Methods" section for definition of estimated ranges: low, mid and high. Created using the R statistical software version 4.0.2^22^.

Given our assumed stationarity of the shape and scale parameters, the nonstationary location parameter is used solely to determine the incremental change to future extreme wave climate. Figure 3 shows the centennial (100 year) change in the fitted nonstationary location parameter for the GEV fit ($\delta {H}_{m0,100}$ in metres) to the GWCM ensemble, for the RCP 8.5 scenario, as described in the “Methods” section. Also shown in Figure 3 are the small differences between the Gumbel and GEV fits which indicates that the addition of a shape parameter has little influence on detecting the incremental change in the location parameter.

We find that the parameters of the two EVDs (Gumbel and GEV) fitted independently to either the RCP4.5 or RCP8.5 ensemble simulations have noticeably similar global patterns (Figs. 2, 3 and S3). The only noticeable difference is that the higher RCP predicts a larger incremental change (positive or negative) in the non-stationary location parameter (Figure S5). We therefore deduce that changes in extreme wave climate behaviour (storminess or intensity) in the RCP forced GWCM simulations is not noticeable beyond an incremental change (positive or negative) to all AEP extremes. The global pattern of incremental change for the two RCP simulations (Figure S5) show some similarity to the rate of change identified by a linear trend fitted to the annual mean (Figure S6), but differs in sign at many sites in the North Pacific and Atlantic Oceans, indicating twenty-first century changes in extreme wave conditions do not necessarily follow the pattern of the mean. The mid-range estimates of $\delta {H}_{m0,100}$ show larger waves at higher latitudes, but also show that subtropical regions could experience a decrease in extreme wave heights. The high-end estimates indicate that extreme waves ($\delta {H}_{m0,100}$) at high latitude could be of the order of 2 m taller by the end of the twenty-first century, but not all models predict an increase in extreme wave heights everywhere. The low-end estimates show that at some sites, even low-end ensemble estimates predict an increase. The sites showing a high model likelihood of an increase are the waters south-west of Tasmania, Australia, the Arabian Sea and the Gulf of Guinea.

**Figure 4:**
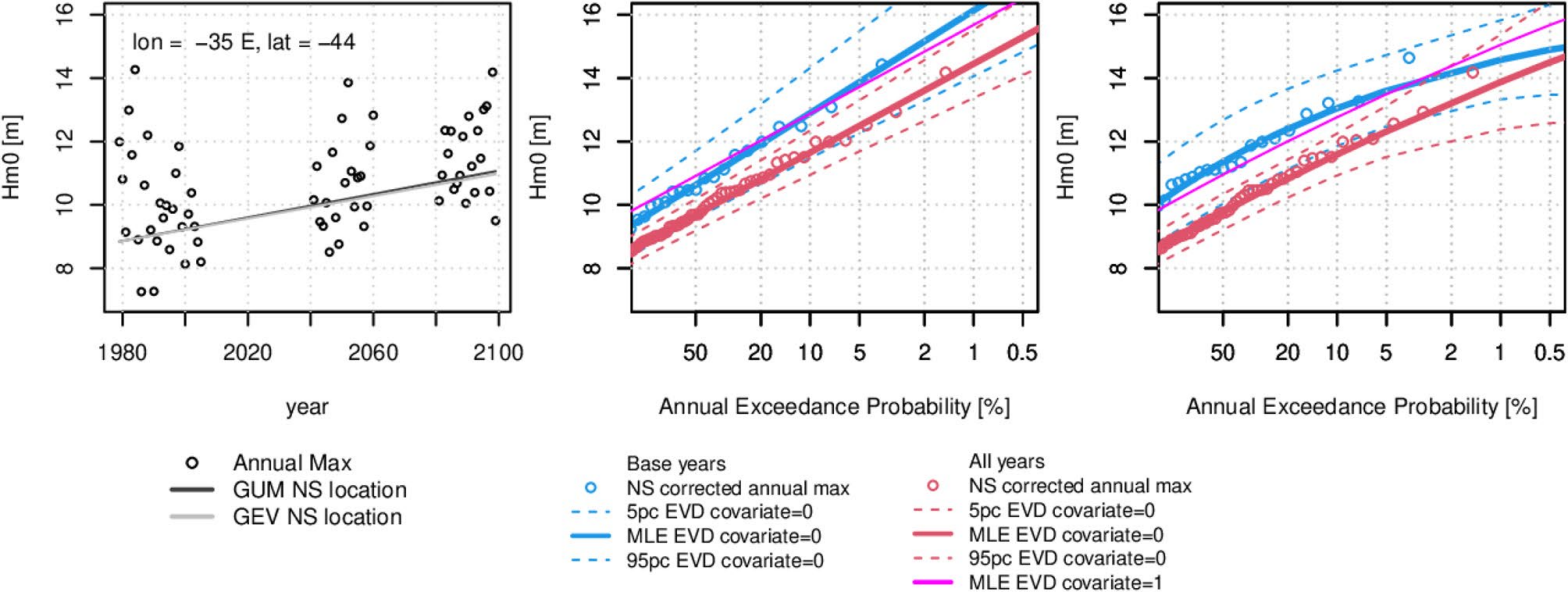
EVD fits to the GFDL-CM3 model output at an example location south of Tasmania, Australia. Left is time series of annual max Hm0 with the trend line of nonstationary (NS) location parameter for the Gumbel and GEV fits. Centre plot is Gumbel EVD for the baseline and full RCP85 periods including the MLE and 90% confidence intervals for the covariate equals zero at 1979 and the MLE for the covariate equals one at 2100. Right is same as the centre but for a GEV EVD. Created using the R statistical software version 4.0.2^22^.

Figure 4 shows the $\delta {H}_{m0}$ trend in the annual maximum events at a location south of Tasmania, Australia where there is a high model likelihood/agreement in an increase in future AEPs (Fig. 3). Also shown in Figure 4 are return level curves (GEV and Gumbel in separate plots) comparing the EVD fitted to 27 year baseline period and the full GWCM period (66 years over the period 1979 to 2100), i.e. comparing the fully non-stationary fitting of two time slices, the shape and scale parameters can be different due to sample size. Fitting the GEV distribution to the baseline periods show a significantly different shape parameter to the full time period, which we believe is due to sensitivity of fit to record length and not climate change^19^, as we show the shape parameter is the same for the two divergent climate scenarios (RCP 4.5 and 8.5; Fig. 2). Further EVD examples for the Arabian Sea and the Gulf of Guinea and West North Atlantic Ocean are provided in Figure S7.

**Figure 5:**
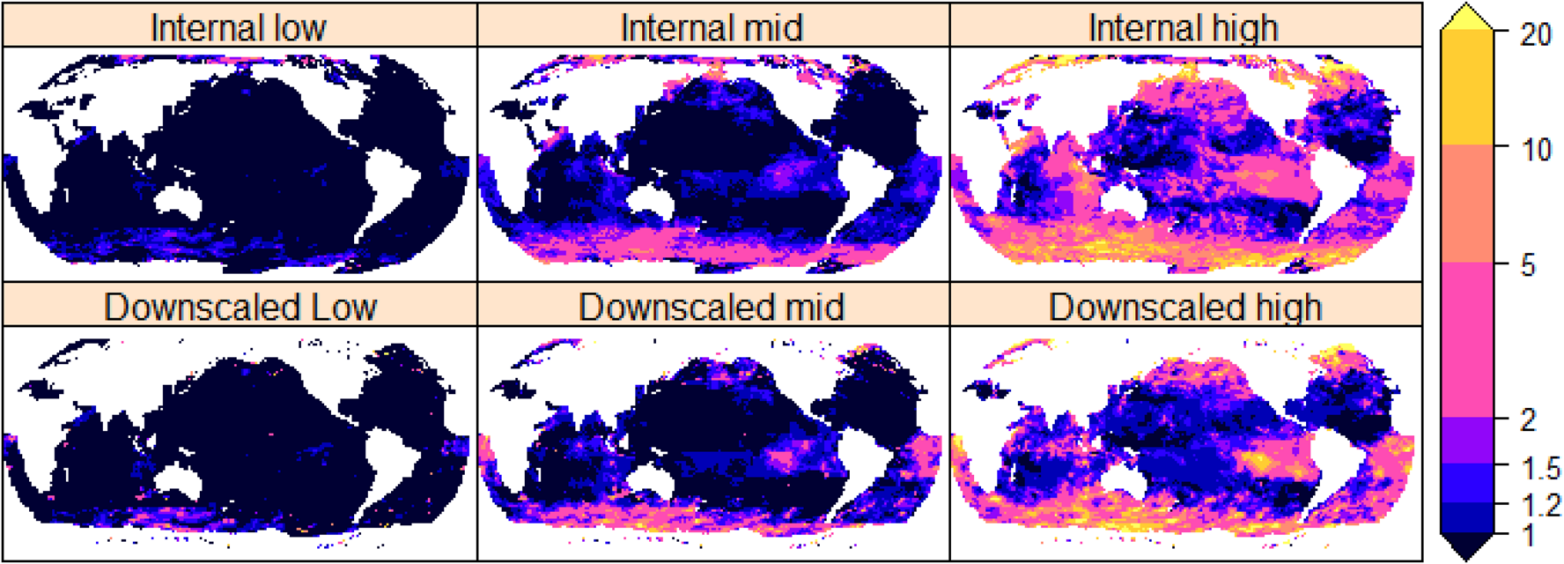
GEV RCP85 amplification of the baseline 1% AEP over a period of 100 years. Log colour scale indicates the amplification factor. Top row: Internal model estimates using GWCM GEV parameters, bottom row: Downscaled estimates using the hindcast GEV parameters with the incremental change to the location parameter ($\updelta {\mathrm{H}}_{\mathrm{m}0}$). Created using the R statistical software version 4.0.2^22^.

The incremental change in location parameter $\updelta {\mathrm{H}}_{\mathrm{m0,100}}$ can be easily used in site specific CF downscaling studies to better identify the future change in the frequency of AEP events (see “Methods” section). The GWCM EVD parameters can be used to estimate the GWCM internally derived (internal) amplification factor. Alternatively, just the change in the location parameter from a GWCM can be used with hindcast EVA parameters to estimate the CF “downscaled” amplification. An amplification factor of two implies that what is a 1% AEP (or 1 in 100 year event) in the baseline climate is expected to become a 2% AEP (or 1 in 50 year event) by the end of the twenty-first century. Figure 5 shows the factor of amplification in exceedance probability for the internal GWCM model GEV parameters, and when $\updelta {\mathrm{H}}_{\mathrm{m0,100}}$ is applied (CF downscaled) to hindcast GEV parameters (see “Methods” section). Here the differences between the spatial variability in the internally derived GWCM amplification and the hindcast downscaled are noticeable, where the change in frequency (amplification) of AEP events is highly sensitive to the spatial variability in the hindcast estimates of EVD shape parameter.

**Figure 6:**
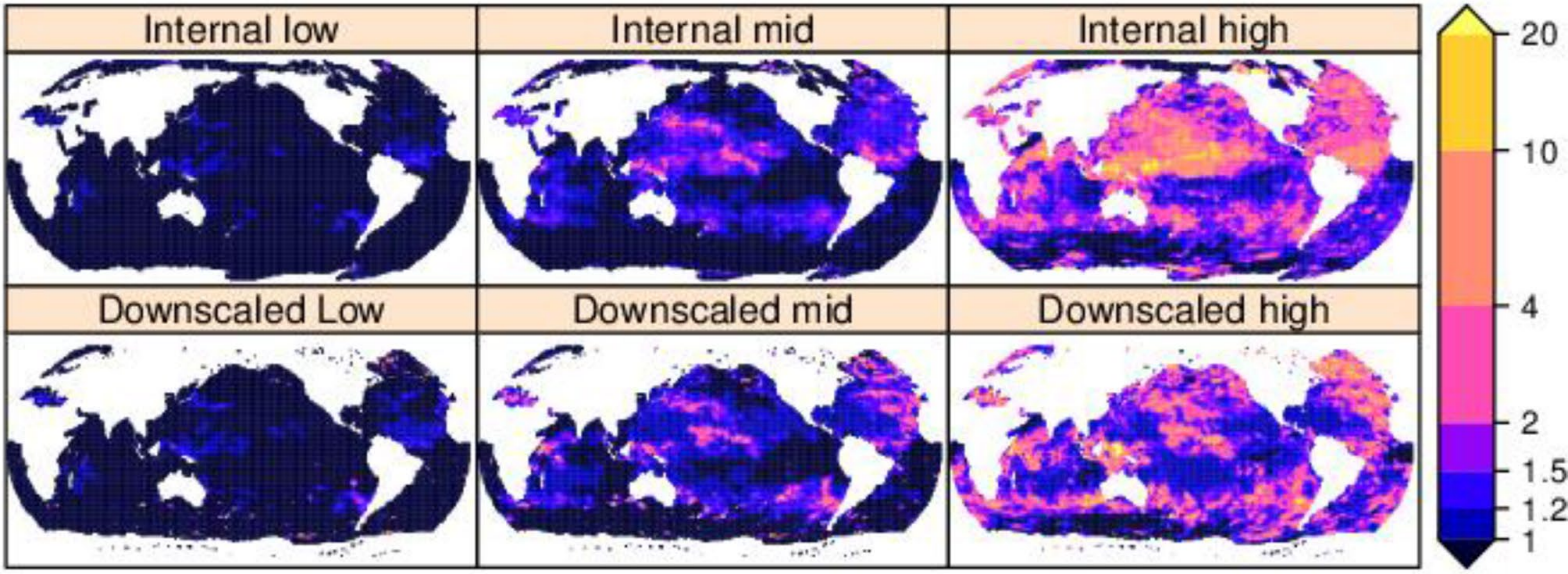
Same as Fig. 5 but for a reduction of the baseline 1% AEP over a period of 100 years. created using the R statistical software version 4.0.2^22^.

Figure 6 shows the factor of reduction in exceedance probability for the internal GWCM model GEV parameters, and when negative $\updelta {\mathrm{H}}_{\mathrm{m0,100}}$ is applied (CF downscaled) to hindcast GEV parameters (see “Methods” section). A reduction factor of two implies that what is a 1% AEP (or 1 in 100 year event) in the baseline climate is expected to become a 0.5% AEP (or 1 in 200 year event) by the end of the twenty-first century. In regions where there is no amplification (Fig. 5) a reduction is shown. The projected change indicates a decrease in tropical waters in all oceans, which could potentially result in reduced flushing time of reef lagoons and impact reef health^31^.

## Discussion

We show that an ensemble of GWCM forced global wave simulations (GWCMs) spans the range of EVD parameters fitted to a detailed hindcast simulation^31,32^. The GEV shape parameter, shown to be sensitive to record length^19^, along with the Gumbel scale parameter, have indistinguishable similarity in spatial pattern for divergent RCP simulations (4.5 and 8.5). Thus, an assumption of stationarity in the shape and scale parameters appears valid for changing climate scenarios. Further consideration should be given to the limitations of a GWCM to generate intense and random storm climates which could eventuate. The ensemble range of the non-stationary fit of the location parameter indicates three regions of high model likelihood of an increase. They are the waters South of Australia, the Arabian Sea, and the Gulf of Guinea. The non-stationary parameter provides an easy to apply CF downscaling method, e.g. compared to dynamic downscaling, to investigate local coastal impacts of episodic events. More detailed investigations where the impacts depend on wave direction or period should consider other more resource-intensive methods, such as dynamic downscaling^33,34^ or multivariate statistical analysis^20,35^. In this study, a limited ensemble of GWCMs that each contain over 60 years of simulation (historical, mid- and end-Century) were used. Larger ensembles are available that could permit a more complete representation of uncertainty^14^. Future studies could broaden the ensemble estimates, provided ensemble members are of sufficient length to obtain more robust parameter estimates. We present a range of models to allow coastal practitioners to consider the uncertainty in the modelling estimates, in understanding the ‘worst case’, ‘best case’ and ‘most likely’ GWCM model projection for both an increase and decrease in extreme wave heights in their impact assessment. GWCMs have shown to exhibit varying levels of bias in the estimate of significant wave height^36^. In this study, the method for measuring the change in the location parameter avoids the requirement for bias correction^36^.

Future work to apply the downscaled changes to coastal impact studies could, for example, revisit investigations into climate driven changes in the contribution of waves to future extreme sea levels^5,37^. Due to the low probability of TC occurrence, future work is required to represent a larger sample of TC extremes, via centennial GWCM or synthetic simulations, to get a better understanding of shape parameter behaviour. The analysis of stationary and non-stationary EVD behaviour in GCMs presented here for wind-driven wave heights could also be considered and tested for other climate variables, such as surface temperatures or wind speeds.

## Methods

All model analysis was conducted and plotting/figures created using the R statistical software version 4.0.2^22^.

### Global spectral wave model data

Globally gridded annual maximum significant wave heights were sourced from a hindcast for the period 1979–2018^32^ and eight GWCM ensemble simulations^26^. The ¼ nautical degree hindcast was regridded onto the 1 nautical degree grid of the GWCM using a bilinear point sample, and all global output projected onto the Robinson projection with the prime meridian centred on the central pacific dateline^38^.

### Extreme value analysis

Gumbel and GEV extreme value distributions were fitted to annual maximum significant wave heights using the ‘ismev’ R package^15^. Nonstationary EVD fitting of the location parameter ($\mu$) was exclusively applied to all GEV fits in this study. A non-stationary change in the location parameter will therefore offset the entire return level curves (Gumbel or GEV) either up or down by a given increment. A covariate (${t}_{c}$) representing the temporal change in the EVD location parameter linearly increased from zero in the year 1979 to one in 2100, ${t}_{c}=\frac{nt}{2100-1979}$, where $nt$ is the number of years to project into the future. The rate of change in location parameter with the time-covariate ($\frac{\Delta \mu }{\Delta {t}_{c}}$) has units m/year and is used to estimate the incremental change in extreme significant wave heights ($\delta {H}_{m0,nt}= \frac{\Delta \mu }{\Delta {t}_{c}}{t}_{c})$ with units m over a number of years ($nt$). The EVD fitting was applied to both the annual maxima of baseline period (1979 to 2004) and the full model period of the hindcast (1979 to 2018) or the GWCMs noncontinuous time slices (baseline spans 1979 to 2004, mid-century spans 2025 to 2045 and end-of-century spans 2080 to 2100).

The factor of amplification in exceedance probability for the Gumbel EVD was derived from^39^ with the sea level rise term ($\delta z$) replaced by the incremental change to location parameter ($\delta {H}_{m0}$). GEV factor of increase in exceedance probability were derived from^2^ with the sea level rise term (${\mu }_{SL}$) replaced by ($\delta {H}_{m0}$). The factor for the reduction in exceedance probability is calculated in the same way, except we use the negative of the incremental change to location parameter ($\delta {H}_{m0}$).

### Estimated ranges: low, mid, and high-end range statistics

We compare the hindcast fitted EVD uncertainty range to the quantile range of the GWCM model ensemble, both representing 90% range of estimates. Hindcast ranges were derived from plus or minus 1.64 times the standard deviation of EVD fitted parameter to represent 90% of EVD fitted uncertainty and the 5, 50 and 95th quantiles. GWCM ranges were calculated from sample quantiles (5 50 and 95th) of the eight GWCM ensemble using the recommended method of Hyndman and Fan^40^.

## Supplementary Information

Supplementary information.

## References

[1] IPCC. *Summary for Policymakers. In: IPCC Special Report on the Ocean and Cryosphere in a Changing Climate [H.-O. Pörtner et al]*. (2019). http://www.ipcc.ch/publications_and_data/ar4/wg2/en/spm.html.

[2] Vitousek S (2017). Doubling of coastal flooding frequency within decades due to sea-level rise. Sci. Rep..

[3] Lambert E, Rohmer J, Le Cozannet G, Van de Wal RSW (2020). Adaptation time to magnified flood hazards underestimated when derived from tide gauge records. Environ. Res. Lett..

[4] Kirezci E (2020). Projections of global-scale extreme sea levels and resulting episodic coastal flooding over the 21st Century. Sci. Rep..

[5] Vousdoukas MI (2018). Global probabilistic projections of extreme sea levels show intensification of coastal flood hazard. Nat. Commun..

[6] Casas-Prat M, Wang X (2020). Projections of extreme ocean waves in the Arctic and potential implications for coastal inundation and erosion. J. Geophys. Res. Ocean..

[7] Melet A (2020). Contribution of wave setup to projected coastal sea level changes. J. Geophys. Res. Ocean..

[8] Meucci A, Young IR, Hemer M, Kirezci E, Ranasinghe R (2020). Projected 21st century changes in extreme wind-wave events. Sci. Adv..

[9] Taebi S (2011). Nearshore circulation in a tropical fringing reef system. J. Geophys. Res..

[10] Udo K, Ranasinghe R, Takeda Y (2020). An assessment of measured and computed depth of closure around Japan. Sci. Rep..

[11] van Vuuren DP (2011). The representative concentration pathways: An overview. Clim. Change.

[12] Hemer MA, Wang XL, Weisssse R, Swail VR (2012). Advancing wind-waves climate science: The COWCLIP project. Bull. Am. Meteorol. Soc..

[13] Morim J, Hemer MA, Cartwright N, Strauss D, Andutta F (2018). On the concordance of 21st century wind-wave climate projections. Glob. Planet. Change.

[14] Morim, J. *et al.* Robustness and uncertainties in multivariate wind-wave climate projections. *Nat. Clim. Chang.* **submitted**, (2019).

[15] Coles S (2001). An Introduction to Statistical Modeling of Extreme Values. Journal of Chemical Information and Modeling.

[16] Gumbel EJ (1941). The return period of flood flows. Ann. Math. Stat..

[17] Jenkinson AF (1955). The frequency distribution of the annual maximum (or minimum) values of meteorological elements. Q. J. R. Meteorol. Soc..

[18] Izaguirre C, Méndez FJ, Menéndez M, Losada IJ (2011). Global extreme wave height variability based on satellite data. Geophys. Res. Lett..

[19] Vanem E (2015). Non-stationary extreme value models to account for trends and shifts in the extreme wave climate due to climate change. Appl. Ocean Res..

[20] Davies G (2017). Improved treatment of non-stationary conditions and uncertainties in probabilistic models of storm wave climate. Coast. Eng..

[21] Wahl T (2017). Understanding extreme sea levels for broad-scale coastal impact and adaptation analysis. Nat. Commun..

[22] R Core Team (2020). R: A Language and Environment for Statistical Computing.

[23] O’Grady J (2019). Downscaling future longshore sediment transport in south eastern Australia. J. Mar. Sci. Eng..

[24] Ekström M, Grose MR, Whetton PH (2015). An appraisal of downscaling methods used in climate change research. Wiley Interdiscip. Rev. Clim. Chang..

[25] Ranasinghe R (2020). On the need for a new generation of coastal change models for the 21st century. Sci. Rep..

[26] Hemer MA, Trenham CE (2016). Evaluation of a CMIP5 derived dynamical global wind wave climate model ensemble. Ocean Model.

[27] Timmermans B, Stone D, Wehner M, Krishnan H (2017). Impact of tropical cyclones on modeled extreme wind-wave climate. Geophys. Res. Lett..

[28] Shimura T, Mori N, Hemer MA (2017). Projection of tropical cyclone-generated extreme wave climate based on CMIP5 multi-model ensemble in the Western North Pacific. Clim. Dyn..

[29] Bloemendaal N (2020). Generation of a global synthetic tropical cyclone hazard dataset using STORM. Sci. Data.

[30] Walsh KJE (2016). Tropical cyclones and climate change. Wiley Interdiscip. Rev. Clim. Chang..

[31] Hemer MA (2017). A revised assessment of Australia’s national wave energy resource. Renew. Energy.

[32] Smith GA (2020). Global wave hindcast with Australian and Pacific Island Focus: From past to present. Geosci. Data J..

[33] Charles E (2012). Climate change impact on waves in the Bay of Biscay, France. Ocean Dyn..

[34] Wandres M, Pattiaratchi C, Hemer MA (2017). Projected changes of the southwest Australian wave climate under two atmospheric greenhouse gas concentration pathways. Ocean Model.

[35] Antolínez JAA (2018). Downscaling changing coastlines in a changing climate: The hybrid approach. J. Geophys. Res. Earth Surf..

[36] Lemos G (2020). On the need of bias correction methods for wave climate projections. Glob. Planet. Change.

[37] O’Grady JG (2019). Extreme water levels for Australian beaches using empirical equations for shoreline wave setup. J. Geophys. Res. Ocean..

[38] Hijmans, R. J. *et al.* Package ‘raster’. *Cran* 1–249 (2020).

[39] Hunter JR (2010). Estimating sea-level extremes under conditions of uncertain sea-level rise. Clim. Change.

[40] Hyndman RJ, Fan Y (1996). Sample quantiles in statistical packages. Am. Stat..

