# Supplementary material for: Projected incremental changes to extreme wind-driven wave heights for the twenty-first century
Source: Sci Rep. 2021 Apr 23;11:8826. doi: 10.1038/s41598-021-87358-w (PMC8065105; doi:10.1038/s41598-021-87358-w)
Supplement: Supplementary file 1 — Supplementary information. [file 41598_2021_87358_MOESM1_ESM.pdf]

Projected incremental changes to extreme wind driven wave heights for the 21st century.

Authors: J G O'Grady[1], M A Hemer[2], K L McInnes[1], C E Trenham[3], A. G. Stephenson[4]. (CSIRO Australia)

1. CSIRO Oceans and Atmosphere, Melbourne Victoria, Australia
2. CSIRO Oceans and Atmosphere, Hobart, Tasmania, Australia
3. CSIRO Oceans and Atmosphere, Canberra, Australian Capital Territory, Australia
4. DATA61 CSIRO, Melbourne, Australia

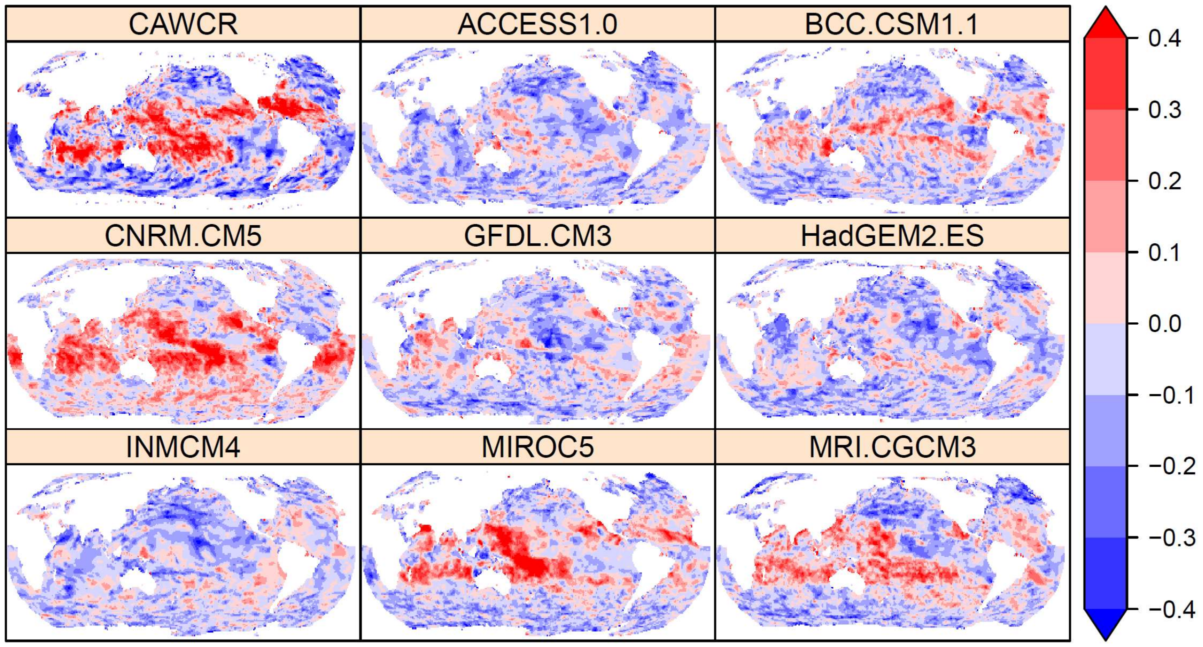

Figure S1 MLE GEV shape parameter for the full period of the CAWCR hindcast and the full period (baseline, mid and end of 21st Century periods) of each GCM RCP 8.5 simulation. created using the R statistical software version 4.0.2 (R Core Team, 2020).

…

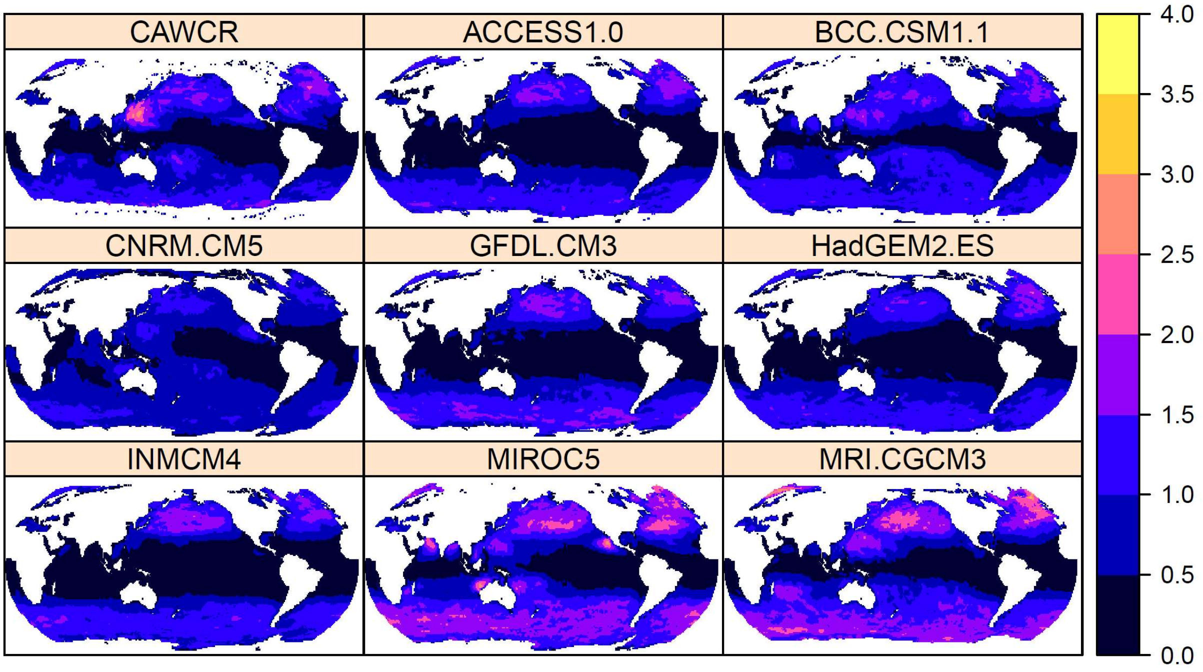

Figure S2 Gumbel scale parameter MLE comparison for the full period of the CAWCR hindcast and the full period (baseline, mid and end of 21st Century periods) of each GCM RCP 8.5 simulation. created using the R statistical software version 4.0.2 (R Core Team, 2020).

…

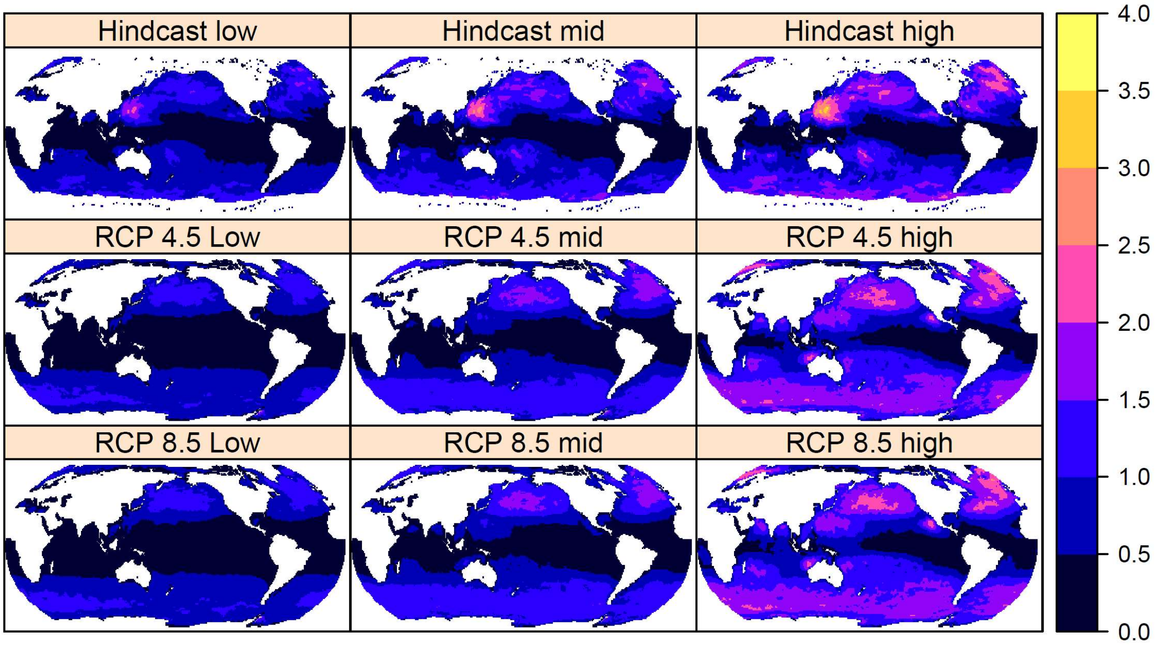

Figure S3 Estimated range of the Gumbel scale parameter for the full period of the CAWCR hindcast and GCM period (baseline, mid and end of 21st Century periods) of the two RCP scenarios. Top row: CAWCR hindcast. Middle: the entire GCM RCP 4.5 period including the baseline, mid and end of 21st Century periods. Bottom: same as middle but for RCP 8.5. created using the R statistical software version 4.0.2 (R Core Team, 2020).

…

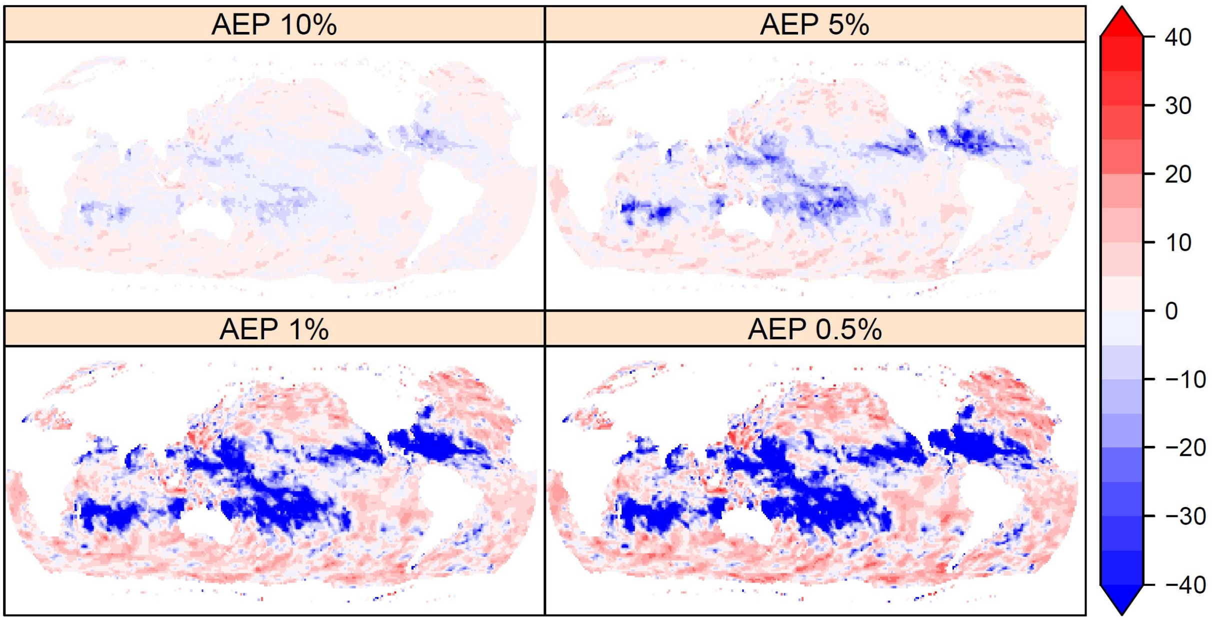

Figure S4 Percentage [%] difference between Gumbel and GEV significant wave height return level AEPs. Calculated as the Gumbel RL less GEV RL divided by the Gumbel RL. created using the R statistical software version 4.0.2 (R Core Team, 2020).

…

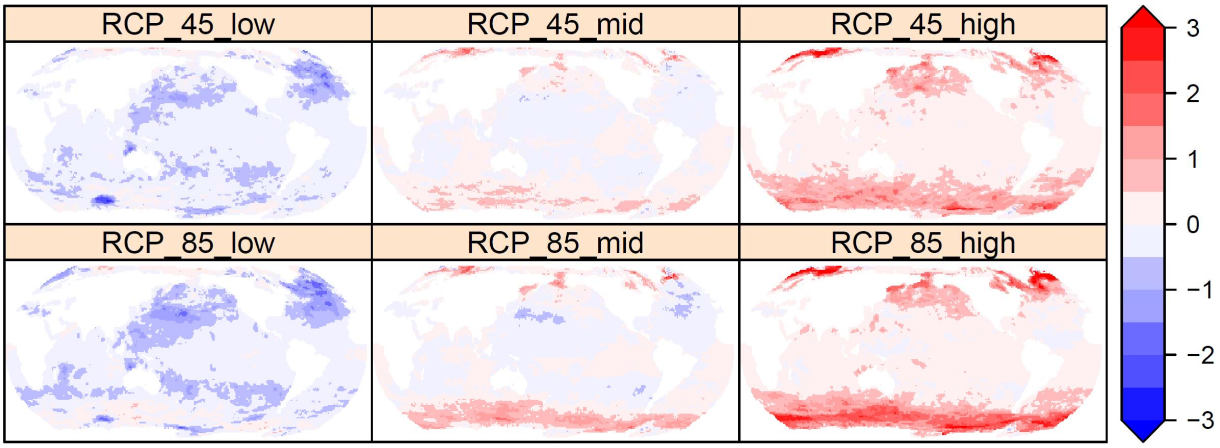

Figure S5 GEV rate of change in location parameter [m] comparison of full GCM RCP simulations over a period of 100 years. Top row shows the 5, 50 and 95% values of the 50% Gumbel fit to 8 GCM RCP45 simulations. Bottom same as middle but for RCP 85. created using the R statistical software version 4.0.2 (R Core Team, 2020).

…

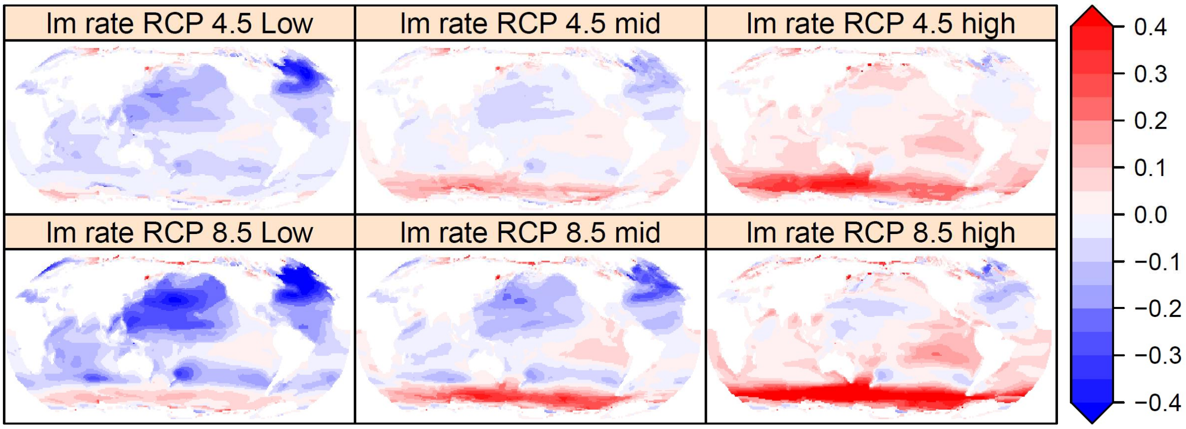

Figure S6 Annual linear model (lm) trend in the anuall mean Hm0 1979-2100. Top row shows the 5, 50 and 95th percentile values of the linear model change [m] over a period of 100 years the 8 GCM RCP45 simulations. Bottom same as middle but for RCP 85. created using the R statistical software version 4.0.2 (R Core Team, 2020).

…

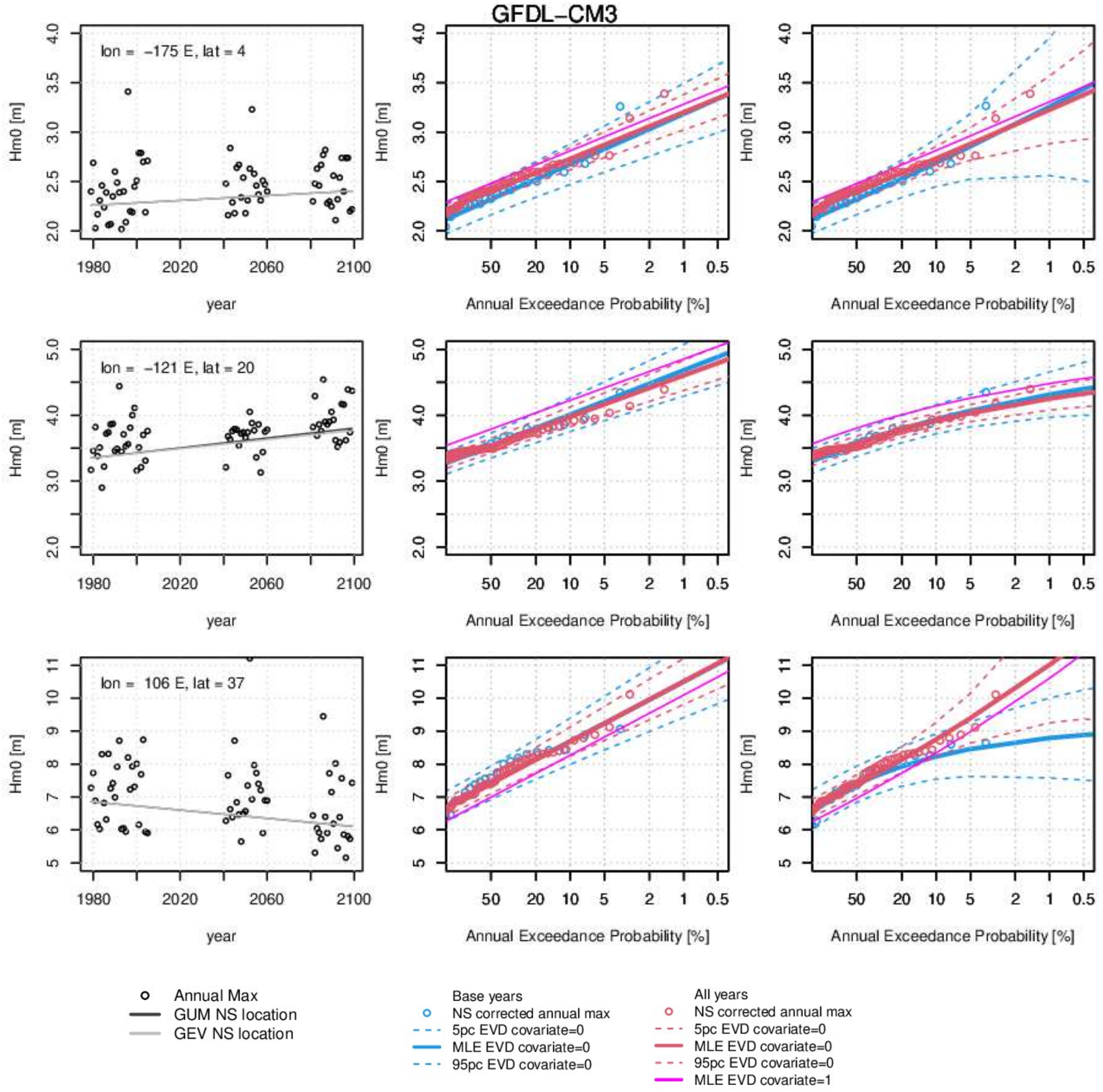

Figure S7 EVD fits to the GFDL-CM3 model output at three example locations. Top row is the Gulf of Guinea, middle row is Arabian Sea and bottom row is West North Atlantic Ocean. Left column is time series of annual max Hm0 with the trend line of nonstationary (NS) location parameter for the GUM and GEV fits. Middle column is Gumbel (GUM) EVD for the baseline and full periods including the MLE and 90% confidence intervals for the covariate equals zero at 1979 and the MLE for the covariate equals one at 2100. Right column is same as the middle column but for a GEV EVD. created using the R statistical software version 4.0.2 (R Core Team, 2020).

…

References

R Core Team. (2020). R: A Language and Environment for Statistical Computing. Vienna, Austria. Retrieved from https://www.r-project.org/
